# A CIC-related-epigenetic factors-based model associated with prediction, the tumor microenvironment and drug sensitivity in osteosarcoma

**DOI:** 10.1038/s41598-023-49770-2

**Published:** 2024-01-15

**Authors:** Bin Yu, Chengkui Geng, Zhongxiong Wu, Zhongzi Zhang, Aili Zhang, Ze Yang, Jiazheng Huang, Ying Xiong, Huiqin Yang, Zhuoyuan Chen

**Affiliations:** 1 Key Laboratory of Tumor Immunological Prevention and Treatment of Yunnan Province, Yan’an Hospital Affiliated to Kunming Medical University, Kunming, Yunnan Province China; 2 Department of Orthopedics of Yan’an Hospital Affiliated to Kunming Medical University, Kunming, Yunnan Province China; 3 Greehey Children’s Cancer Research Institute, University of Texas Health Science Center at San Antonio, San Antonio, TX USA

**Keywords:** Bone cancer, Cancer microenvironment, Tumour immunology, Epigenomics

## Abstract

Osteosarcoma is generally considered a cold tumor and is characterized by epigenetic alterations. Although tumor cells are surrounded by many immune cells such as macrophages, T cells may be suppressed, be inactivated, or not be presented due to various mechanisms, which usually results in poor prognosis and insensitivity to immunotherapy. Immunotherapy is considered a promising anti-cancer therapy in osteosarcoma but requires more research, but osteosarcoma does not currently respond well to this therapy. The cancer immunity cycle (CIC) is essential for anti-tumor immunity, and is epigenetically regulated. Therefore, it is possible to modulate the immune microenvironment of osteosarcoma by targeting epigenetic factors. In this study, we explored the correlation between epigenetic modulation and CIC in osteosarcoma through bioinformatic methods. Based on the RNA data from TARGET and GSE21257 cohorts, we identified epigenetic related subtypes by NMF clustering and constructed a clinical prognostic model by the LASSO algorithm. ESTIMATE, Cibersort, and xCell algorithms were applied to analyze the tumor microenvironment. Based on eight epigenetic biomarkers (SFMBT2, SP140, CBX5, HMGN2, SMARCA4, PSIP1, ACTR6, and CHD2), two subtypes were identified, and they are mainly distinguished by immune response and cell cycle regulation. After excluding ACTR6 by LASSO regression, the prognostic model was established and it exhibited good predictive efficacy. The risk score showed a strong correlation with the tumor microenvironment, drug sensitivity and many immune checkpoints. In summary, our study sheds a new light on the CIC-related epigenetic modulation mechanism of osteosarcoma and helps search for potential drugs for osteosarcoma treatment.

## Introduction

Osteosarcoma (OS) is one of the most common bone tumors occurring predominantly in children and adolescents. Another prone population is the people with age > 60 years, which is generally correlated with Paget’s disease of bone^1,2^. As a solid tumor with a high level of heterogeneity, osteosarcoma possesses a complex immune environment. Macrophages are the primary immune cells surrounding the malignant cells, and other innate immune cells, such as T-lymphocytes, dendritic cells, mast cells, neutrophils, and B cells, also exhibit a certain level of infiltration^3,4^. However, T cells may not be present or may be suppressed by various mechanisms involving the lack of T-cell clonal diversity and low expression of programmed death ligand (PDL) in the tumor^5,6^. Due to this specific immune microenvironment, osteosarcoma generally is considered a “cold” tumor, which results in a poor response to immunotherapy^7^. Immunotherapy is an emerging and promising therapy method utilizing the immune system to battle cancer^8^. Immune checkpoint blockade (ICB) is typical of immunotherapy, which achieves unprecedented advances in cancer treatment. PD-L1, a vital target for ICB, was found expressed in osteosarcoma cell lines and correlated with drug resistance of osteosarcoma. In addition, anti-PD1 and anti-PD-L1 therapy exhibited good effectiveness in an osteosarcoma mouse model^9^. Hence, ICB is considered a potential promising therapy for osteosarcoma patients. However, the application of ICB therapy in clinical treatment encounter some obstacles. Only a small subset of patients benefit from ICB and immune-related adverse events (irAEs) occur in partial patients^10^. In order to improve the effectiveness and application extent of immunotherapy, there is an urgent need to realize the regulation mechanism of anti-cancer immunity. The cancer immunity cycle (CIC) is defined as a series of stepwise events required for anti-cancer immunity, and the therapy targeting these steps is a strategy favoring immunotherapy^11,12^. Firstly, dendritic cells capture cancer antigens released from tumor cells and present them to naïve T cells that prime and activate effector T cells. Then the effector T cells infiltrate the tumor microenvironment (TME) and kill tumor cells. Finally, cancer antigens are released again and the next cycle starts^13^. The dysregulation of CIC can result in the immune escape and survival of tumor cells^13,14^. In order to accelerate the development and application of immunotherapy in osteosarcoma, it is essential to explore the regulatory mechanism of CIC.

Epigenetic factors affect tumors not only by regulating the activities of tumor cells but also through impacting TME^15^. Epigenetic modification, comprising DNA methylation, histone modification, nucleosome remodeling, RNA modification, and non-coding RNA regulation, is a critical regulation mechanism in phenotypic alteration without changes in DNA sequences^16,17^. Osteosarcoma displays global hypomethylation and focal hypermethylation at CpG islands^18^. Abnormal DNA methylation is able to regulate RNA expression levels. For instance, TSSC3, a pro-apoptosis gene, is silenced in osteosarcoma cells due to the hypermethylation at promoter regions^19^. Histone modification refers to the post-translational modification of histone protein tails, which can impact nucleosome dynamics, and transcription^20^. Many enzymes modifying histone proteins are involved in the tumorigenesis of osteosarcoma. Histone demethylase KDM4A is upregulated in osteosarcoma and alleviates tumor cell ferroptosis through mediating H3K9me3 demethylation in the promoter region of SLC7A11^21^. Histone acetyltransferase HBO1 is increased in osteosarcoma and serves as an oncogene^22^. RNA modification and non-coding RNA contribute to osteosarcoma progression via regulating the translation of mRNA to proteins. N6-methylation (m6A) is the most common RNA modification in tumors, and various m6A related enzymes play a regulatory role in osteosarcoma. ALKBH5, a type of m6A eraser, is found to inactive STAT3 pathway in osteosarcoma through decreasing m6A modification of SOCS3^23^. These enzymes also can interact with non-coding RNAs. PVT1, a vital oncogenic long noncoding RNA (lncRNA), is regulated by ALKBH5 via a m6A-depended manner^24^. Interestingly, epigenetic regulation also plays a vital role in the immune system, and the therapy targeting epigenetic modulation is considered a meaningful complement to immunotherapy^25^. It was found that immunomodulatory pathway genes were concentrated in late-replicating partial methylation domains with DNA methylation loss^26^. A pan-cancer analysis also revealed that tumor immunogenicity was inversely associated with methylation aberrancy^27^. Wholescale epigenetic remodeling was observed in exhausted T cells that weakens the response to immunotherapy^28^. Epigenetic therapy not only directly inhibits tumors, but also assists the function of immunotherapy^29^. Several epigenetic drugs have been approved for anti-cancer treatment by the Food and Drug Administration (FDA) of America, including DNMT inhibitors, HDAC inhibitors, and so on^30^. Many studies found that a part of epigenetic drugs can work as inducers of tumor immunogenic cell death (ICD)^31^. Besides, HDAC inhibitors were found to increase the expression levels of CTLA-4, GITR, and PD-1 in Treg cells^32^. Although increasing clinical data suggest the great potential of the combination of epigenetic drugs and immunotherapy, there remain a series of obstacles in the clinical implementation of this combination therapy, such as the ubiquitous distribution of epigenetic targets in normal and tumor cells^33^.

In osteosarcoma, the interaction between epigenetic modulation and immune response remains largely unexplored, and the value of combination therapy including epigenetic modulators and immunotherapy requires further evaluation. Hence, we explored the correlation between CIC events and epigenetic factors in osteosarcoma, and identified two molecular subtypes with different epigenetic and immune characteristics, based on which a clinical risk model was constructed to predict the clinical outcomes of osteosarcoma patients.

## Material and methods

### Data source and process

The current study collected 801 epigenetic factor encoding genes (Table S1) from EpiFactors Database (https://epifactors.autosome.org/). In this database, epigenetic factors are defined as proteins and lncRNAs involved in epigenetic regulation. Protein factors consist of modulators of chromatin, cofactors forming complex with epigenetic factors, histones and corresponding variants, protamines, histone chaperones, and DNA/RNA modification regulators^34^.

Two cohort datasets were included in this study. The RNA expression data and corresponding clinical data of 95 patients were downloaded from the TARGET database (https://ocg.cancer.gov/programs/target). The GSE21257 dataset (Platform: GPL10295), containing gene expression data and clinical data of 53 patients, was downloaded from the Gene Expression Omnibus public database (GEO: https://www.ncbi.nlm.nih.gov/geo/). The two datasets were merged and then applied to clustering. The batch effect was corrected by the “sva” R package. In the construction of prognostic model, the TARGET dataset was set as a train cohort, and the GSE21257 dataset was used as a validation cohort. The RNA expression data of 148 samples was presented in Table S2, and corresponding clinical information was shown in Table S3.

### Identification of CIC-related epigenetic factors with prognostic value

Using Tracking Tumor Immunophenotype database (TIP: http://biocc.hrbmu.edu.cn/TIP/), CIC score, an indicator to represent the activity levels of CIC steps, was calculated based on RNA expression data of 148 osteosarcoma samples (Table S4). TIP database provides a user-friendly web tool to evaluate and visualize the activity of anticancer immunophenotypes^35^. The correlations between CIC score and epigenetic factors were estimated by Pearson test. The correlations with *P* < 0.05 and |R|> 0.3 were considered significant, which were shown in Table S5 and were visualized using Cytoscape 3.8. We then screened the CIC-related epigenetic factors that were associated with prognosis by univariate Cox analysis. The epigenetic factors with *P* < 0.05 were considered significant (Table S6).

### Molecular subtypes of osteosarcoma

In this study, non-negative matrix factorization (NMF) was applied to clustering the molecular subtypes of osteosarcoma samples. NMF R package was used in the clustering, where the method was set as “brunet”, the rank was set as 2–10, and the iteration number was set as 1000. The optimal rank was determined by cophenetic, dispersion and silhouette indicators. Kaplan–Meier survival curves were drawn to evaluate the prognostic value of this clustering. Furthermore, principal component analysis (PCA) was performed to validate if this clustering method could distinguish osteosarcoma samples with different molecular features. PCA is an unsupervised learning method that can transform high-dimensional data into fewer dimensions, which works for capturing features^36^.

### Functional and pathway enrichment analysis

In order to deeply analyze the differences between osteosarcoma samples from different molecular subtypes, functional and pathway enrichment analysis were performed based on the Kyoto encyclopedia of genes and genomes (KEGG) database and the Gene ontology (GO) database. Gene set variation analysis (GSVA) was applied to explore the enriched pathways based on the KEGG database. Gene set enrichment analysis (GSEA) was applied to explore the functional difference based on the GO database. Enrichment analysis was performed in R software, and R packages “GSVA” and “clusterProfiler” were adopted in the analysis.

### The TME landscape of molecular subtypes of osteosarcoma

The TME landscape of osteosarcoma was portrayed by “estimate”, “cibersort” and “xCell” R packages. Firstly, using “estimate” R package, we calculated stromal score, immune score and tumor purity, which reflected the content of stromal cells, immune cells, and tumor cells respectively. Then, we calculated the priority of 22 immune cells for each sample by “cibersort” R package. Finally, we calculated the enrichment score for 64 immune cells and stromal cells in osteosarcoma via “xCell” R package. The abbreviations of these cell types were provided in the “Abbreviation Section”.

In addition, we compared RNA expression levels of immune checkpoint genes in different molecular subtypes and evaluated immune features for each sample. Immune checkpoint genes were summarized by some recent studies^37,38^. The levels of immune features were estimated by single sample gene set enrichment analysis (ssGSEA).

### Construction and validation of clinical risk model and nomogram

Least absolute shrinkage and selection operator regression (LASSO) is a regression analysis method which can be used to exclude irrelevant variables and consequently downscale data. In the current study, LASSO-Cox regression was applied to screen critical genes and construct a clinical prognostic model. The TARGET cohort (n = 95) was set as a train cohort, and the GSE21257 cohort (n = 53) was set as a validation cohort. The optimal value of tuning parameter (lamda) was determined by ten-time cross validation using minimum criteria. The risk score was calculated by the formula: $Risk\;Score = \mathop \sum \limits_{i} Coefficient \left( i \right) \times Expression\;of\;gene \left( i \right)$. According to the median value of risk score (− 0.0835), patients were divided into a high risk group and a low risk group. Kaplan–Meier curve and Receiver operating characteristic curve (ROC) were plotted to identify the prognostic value of the risk score.

To predict 1-, 3-, and 5- years survival probabilities, we generated a nomogram using a R package “rms”. Risk score and three clinical features were adopted in the construction of a nomogram. Clinical features included age, gender, and metastasis status. Correction curves were plotted to compare prediction accuracy between the observed and model-predicted outcomes. Furthermore, ROC was performed to evaluate the efficiency of the nomogram.

### Comprehensive analysis for the role of risk score in osteosarcoma

In order to comprehensively understand the role of risk score in osteosarcoma, we explored the correlations of risk score with clinical characteristics, immune characteristics, drug sensitivity, and response to immunotherapy.

The clinical characteristic information of osteosarcoma includes age, gender, histologic response, and metastasis status, among which the information of histologic response is incomplete. Immune characteristics were evaluated using the infiltration of immune cells, and the expression levels of immune modulator genes (immune inhibitor genes and immune stimulator genes). The immune modulator genes were summarized by the TISIDB database (http://cis.hku.hk/TISIDB/index.php^39^. The drug sensitivity was defined by the half maximal inhibitory concentration (IC50). The R package “oncoPredict” provides an algorithm to estimate IC50 of drugs based on the gene expression data, which was used in this study to calculate IC50 of drugs for every osteosarcoma patient using drug data from Genomics of Drug Sensitivity in Cancer (GDSC) as train data. The lower IC50 means higher drug sensitivity. Tumor inflammation signature score (TIS) and Immunophenoscore (IPS) were applied to indicate the response to immunotherapy. IPS is a scoring system used to demonstrate immune function, including antigen presentation, effector cells, checkpoints, and suppressor cells. IPS consists of four categories, MHC molecules IPS (MHC_IPS), immunomodulators IPS (CP_IPS), effector cells IPS (EC_IPS), and suppressor cells IPS (SC_IPS), which were calculated based on the RNA expressions of corresponding biomarkers^40^. In our study, the TIS score was calculated using GSVA tools, and the IPS was calculated through “IOBR” R package.

### Statistical analysis

All statistical analysis in our study was performed in R software (version 4.1.3). The prognostic values of CIC-related epigenetic factors were estimated using univariate Cox analysis. The Wilcoxon test was used to compare the differences between the two molecular subtypes, and the differences in clinical features between high and low risk groups. Chi-squared test was used to check if there was a significant difference of risk distribution within groups with different clinical features. Spearman correlation coefficients were employed to evaluate the associations between immune characteristics, drug sensitivity, response to immunotherapy, and risk score.

## Result

### Identification of CIC-related epigenetic factors with prognostic value in osteosarcoma

**Figure 1:**
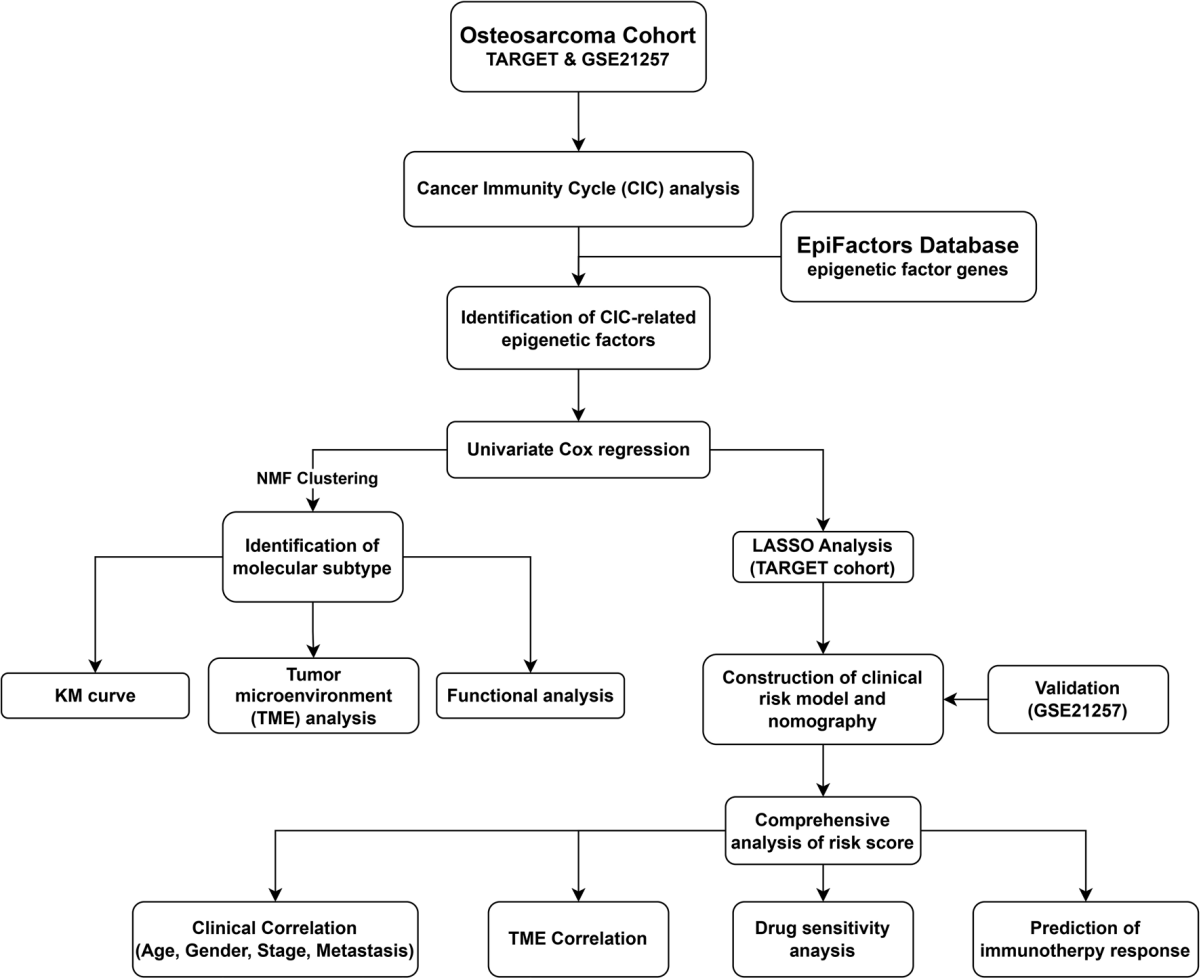
The flowchart of this study.

Figure 1 presents the design of our research. Firstly, we identified CIC-related epigenetic factors with prognostic value, and then we selected significant genes to cluster osteosarcoma and construct a clinical risk model. The clinical information of 148 patients from the TARGET cohort and the GSE21257 cohort were summarized in Table 1.

**Table 1:** The clinical information of TARGET cohort and GSE21257 cohort.

	TARGET_OS (N = 95)	GSE21257 (N = 53)	Overall (N = 148)
Sex			
Male	55 (57.9%)	34 (64.2%)	89 (60.1%)
Female	40 (42.1%)	19 (35.8%)	59 (39.9%)
Age (years)			
Mean (SD)	15.4 (5.32)	18.7 (12.2)	16.6 (8.56)
Median [Min, Max]	14.8 [3.56, 39.9]	16.7 [3.08, 79.0]	15.3 [3.08, 79.0]
Follow-up to main event (years)			
Mean (SD)	4.00 (3.00)	5.71 (4.94)	4.62 (3.88)
Median [Min, Max]	3.33 [0.203, 16.0]	3.75 [0.333, 20.5]	3.48 [0.203, 20.5]
Status			
Alive	57 (60.0%)	30 (56.6%)	87 (58.8%)
Dead	38 (40.0%)	23 (43.4%)	61 (41.2%)
Metastasis			
Yes	23 (24.2%)	34 (64.2%)	57 (38.5%)
No	72 (75.8%)	19 (35.8%)	91 (61.5%)
Histologic response			
Stage 1/2	28 (29.5%)	29 (54.7%)	57 (38.5%)
Stage 3/4	16 (16.8%)	18 (34.0%)	34 (23.0%)
Unknown	51 (53.7%)	6 (11.3%)	57 (38.5%)

**Figure 2:**
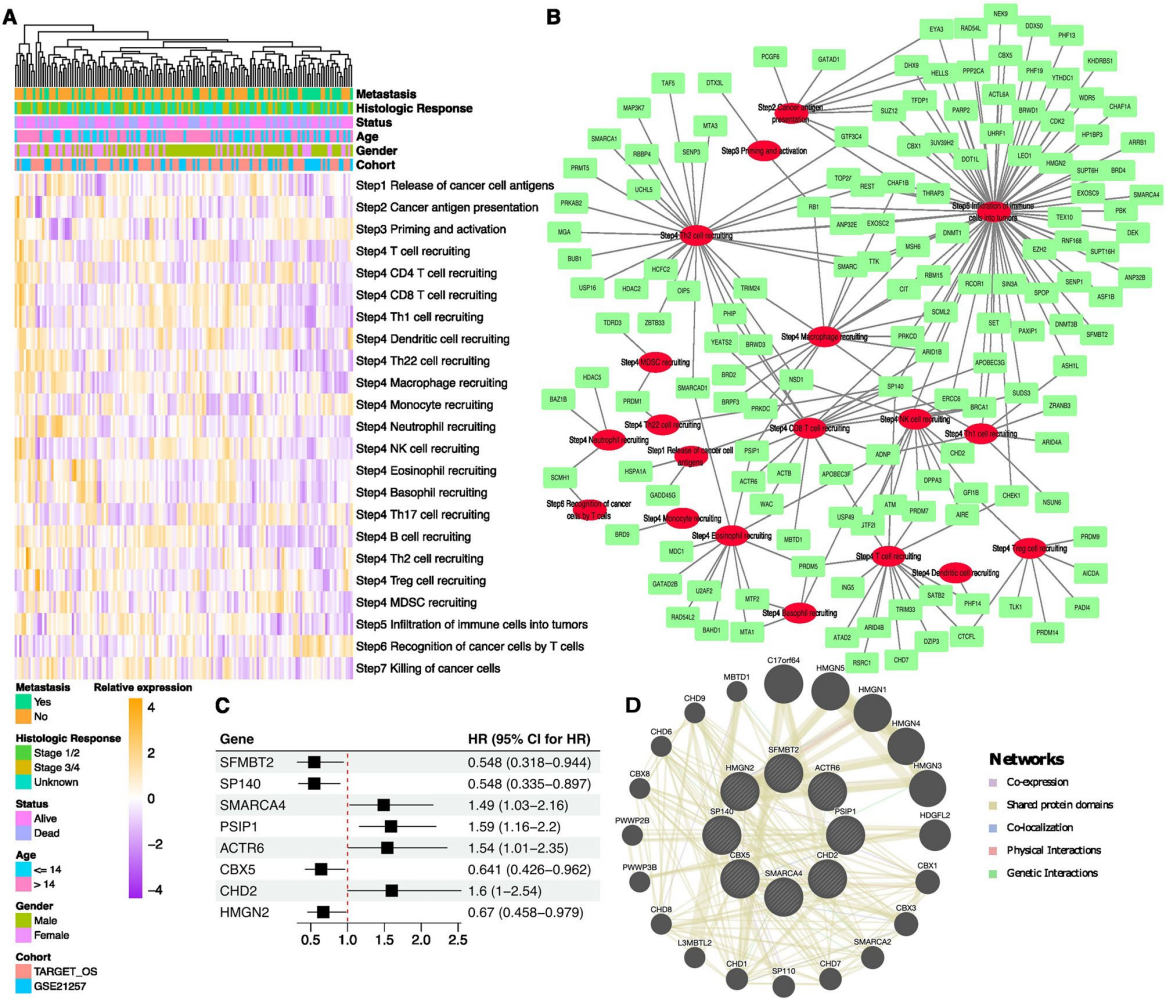
Identification of CIC-related epigenetic factors with prognostic value. (**A**) The heatmap of CIC score of osteosarcoma samples. (**B**) The network diagram of CIC events and epigenetic factors. Red represented CIC events, and green represented epigenetic factors. (**C**) The forest plot of eight epigenetic factors with prognostic value. (**D**) The network plot of epigenetic factors with prognostic value using the GeneMANIA online tool.

CIC steps were quantified by the TIP web tool in the form of a score, which is exhibited in Fig. 2A. Based on the CIC score and RNA expression data, the correlation between CIC steps and epigenetic factors was identified. In total, 149 epigenetic factors were identified associated with CIC (*P* < 0.05, |R|> 0.3), which were presented in Fig. 2B. Then we further screened prognosis related genes among CIC-related epigenetic factors. Finally, eight epigenetic factors were found highly correlated with prognosis. As shown in Fig. 2C, SFMBT2, SP140, CBX5, and HMGN2 were identified as protective factors, whereas SMARCA4, PSIP1, ACTR6, and CHD2 were identified as risk factors. An interaction network for these epigenetic factors was constructed. Eight CIC-related factors with prognostic value were surrounded by 20 genes, among which eight genes exhibited high correlation with CHD family genes (CHD1, CHD6, CHD7, CHD8, CHD9), CBX family genes (CBX1, CBX3, CBX8) and HMGN family genes (HMGN1, HMGN3, HMGN4) (Fig. 2D).

### Identification and comprehensive analysis of molecular subtypes of osteosarcoma

**Figure 3:**
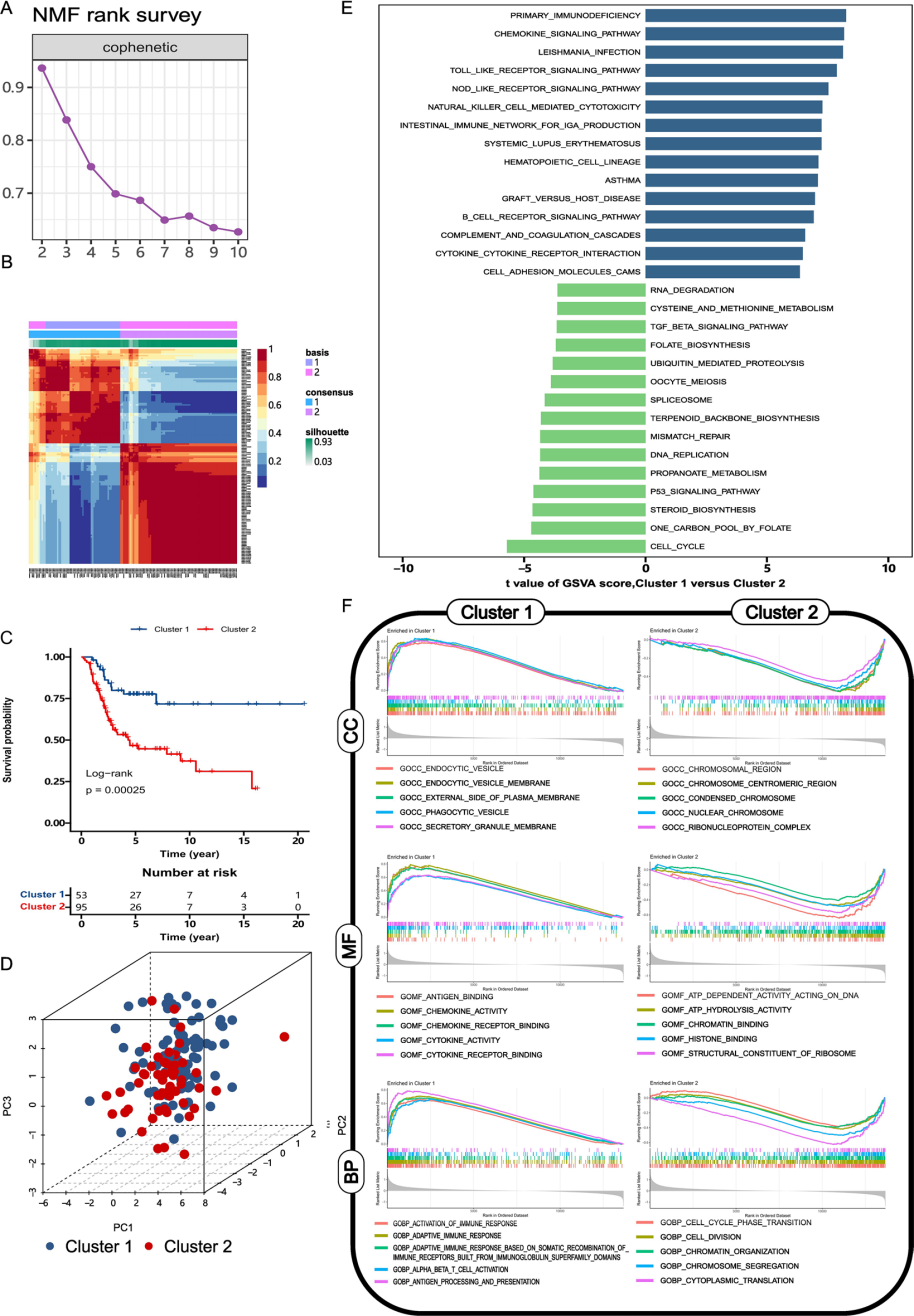
Molecular clustering of osteosarcoma based on CIC-related epigenetic factors. (**A**, **B**) The rank curve and heatmap of NMF clustering. (**C**) KM curve of osteosarcoma patients in different molecular subtypes. (**D**) 3D dot plot of PCA analysis. (**E**) The plot of GSVA analysis based on the KEGG database. The blue columns present the top 15 KEGG pathways with the highest t-value, and the green columns present the top 15 KEGG pathways with the lowest t-value. (**F**) The result of GSEA based on the GO database. The top 5 terms of cellular component (CC), molecular function (MF), and biological process (BP) enriched in Cluster 1 and Cluster 2 were presented.

NMF model was used to cluster osteosarcoma patients. Rank 2 was selected as the optimal cluster number as it exhibited the most significant decrease in the cophenetic correlation coefficient (Fig. 3A). 148 samples were divided into 2 subtypes. Cluster 1 contains 53 patients, and cluster 2 contains 95 patients. As shown in Fig. 3B, this clustering could distinguish osteosarcoma samples. The patients in cluster 1 showed better prognosis compared with cluster 2 (Fig. 3C, *P* < 0.001). Finally, PCA was applied to validate the accuracy of this clustering. 3D dot plot presented that the patients of osteosarcoma were clearly separated (Fig. 3D). The subtype information is presented in Table S7.

To reveal the functional difference between cluster 1 and cluster 2, GSVA and GSEA analysis were performed. GSVA presented that the molecular subtype of osteosarcoma mainly differed in immune related pathways. “Primary immunodeficiency”, “chemokine signaling pathway” and “leishmania infection” were upregulated in cluster1, whereas “cell cycle”, “one carbon pool by folate” and “steroid biosynthesis” were upregulated in cluster 2 (Fig. 3E). The top 5 terms of cellular component (CC), molecular function (MF), and biological process (BP) enriched in cluster 1 and cluster 2 were shown in Fig. 3F. The total results of GSVA and GSEA were presented in Table S8 and S9 respectively.

**Figure 4:**
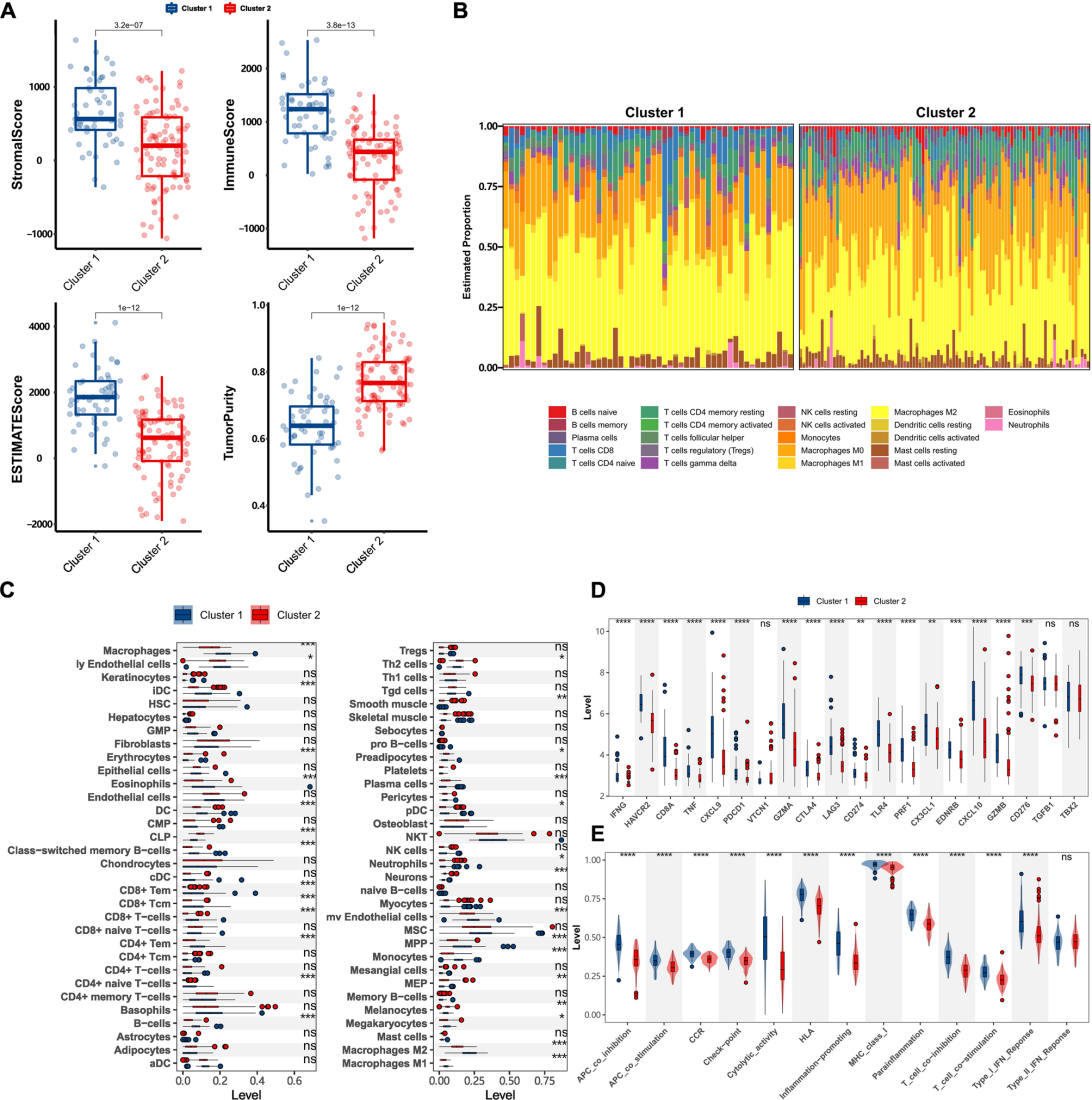
The TME landscape in osteosarcoma. (**A**) Difference analysis of stromal score, immune score, ESTIMATE score, and tumor purity in cluster 1 and cluster 2. (**B**) The abundance of 22 immune cells in 148 osteosarcoma samples based on the “CIBERSORT” algorithm. The length of the bar plot represents the relative abundance of immune cells in each sample. (**C**) Difference analysis of 64 stromal and immune cells in cluster 1 and cluster 2 based on “xCell” algorithm. (**D**) Different expression of immune checkpoint genes in cluster 1 and cluster 2. (**E**) Difference analysis of immune features in cluster 1 and cluster 2. *P < 0.05, **P < 0.01, ***P < 0.001, ****P < 0.0001.

The TME landscape of osteosarcoma samples were analyzed by “ESTIMATE”, “Cibersort” and “xCell” algorithms. “ESTIMATE” algorithm provided stromal score, immune score, ESTIMATE score, and tumor purity, which were exhibited in Fig. 4A. Stromal score, immune score and ESTIMATE score were higher in cluster 1, whereas tumor purity was higher in cluster 2. Figure 4B shows the abundance of 22 immune cells in 148 osteosarcoma samples. As shown in this figure, compared with other immune cells, M2 and M0 macrophages were the immune cells with the highest proportion. Based on the result of the “xCell” algorithm, 64 stromal cells and immune cells were compared between cluster 1 and cluster 2. 29 cells presented differences between cluster 1 and cluster 2 (Fig. 4C). In order to further explore the differences of molecular subtypes in the immune system, immune checkpoint genes, and immune features were researched. 17 immune checkpoint genes were differentially expressed in molecular subtypes, among which IFGN, HAVCR2, CD8A, TNF, PDCD1, VTCN1, GZMA, GZMB, CTLA4, LAG3, CD274, TLR4, PRF1, CX3CL1, CXCL9, CXCL10, EDNRB and CD276 were the most significant genes (*P* < 0.0001) (Fig. 4D). Except for type II IFN response, 12 immune features exhibited differences between cluster 1 and cluster 2 (Fig. 4E). The results of “ESTIMATE”, “Cibersort”, “xCell” and immune features were exhibited in Table S10.

### The construction and validation of clinical risk prognostic model

**Figure 5:**
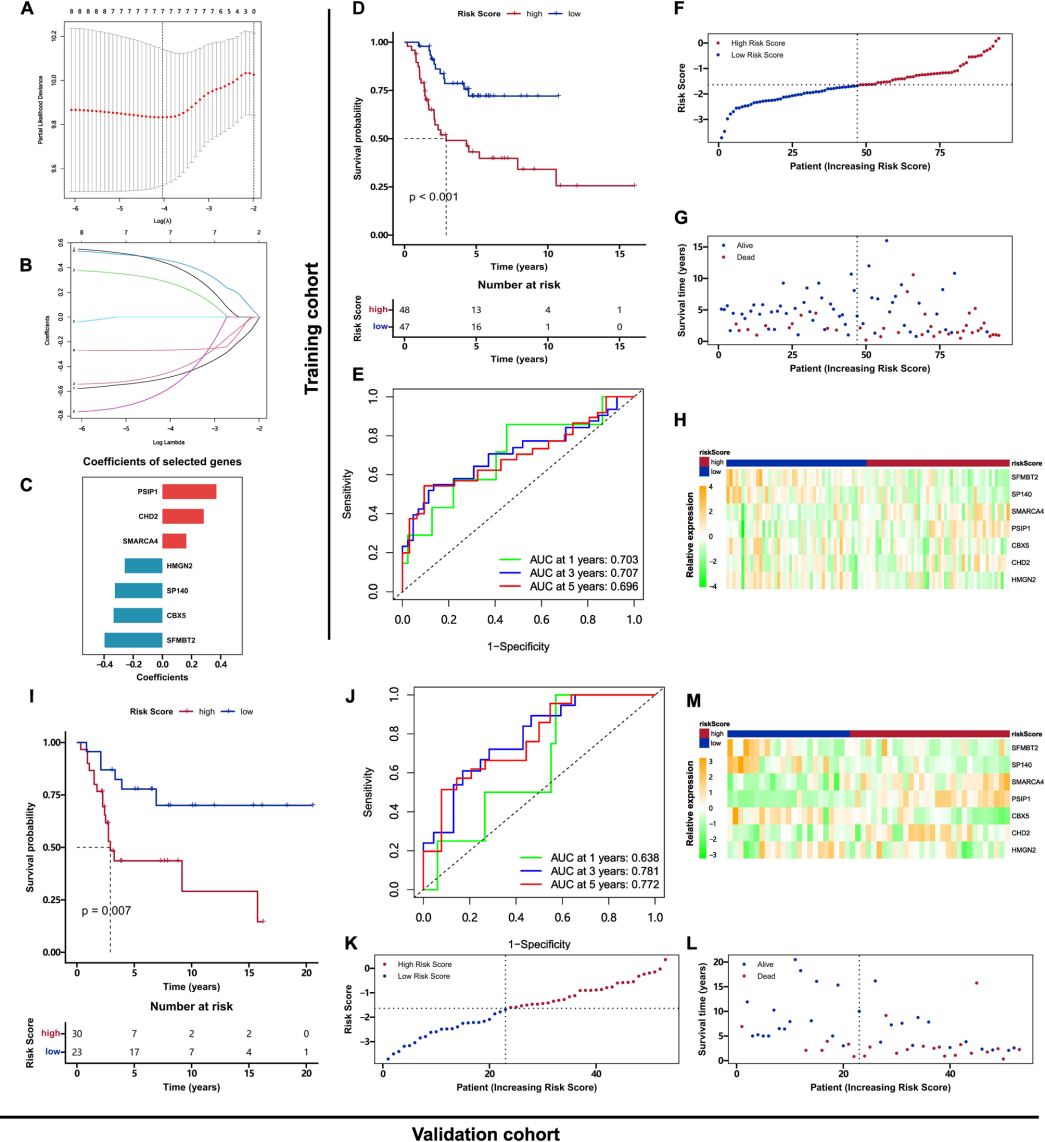
The construction and validation of the clinical risk prognostic model. (**A**) Ten-fold cross-validation for tuning parameter selection in the LASSO model. (**B**) LASSO coefficient profiles of the CIC-related TFs with prognostic value. (**C**) The coefficients of selected genes. Red represented coefficient > 0, and blue represented coefficient < 0. (**D**) Kaplan–Meier curve plot of overall survival for patients from the TARGET cohort. (E) The 1-, 3- and 5- year ROC plots for the TARGET cohort. (F–H) Distributions of risk score, survival status, and RNA expression profile of selected TFs in TARGET cohort. (**I**) Kaplan–Meier curve plot of overall survival for patients from the GSE21257 cohort. (**J**) The 1-, 3-, 5- year ROC plots for the GSE21257 cohort. (K-M) Distributions of risk score, survival status, and RNA expression profile of selected TFs in the GSE21257. The color in heatmaps (H & M) represents the relative RNA expression.

Based on the RNA expression data and clinical information of the TARGET cohort, the LASSO Cox regression algorithm was applied to extract the most critical genes. Seven epigenetic factors were identified as optimal gene signatures, including PSIP1, CHD2, SMARCA4, HMGN2, SP140, CBX5, and SFMBT2 (Fig. 5A–C). Among seven factors, PSIP1, CHD2, and SMARCA4 were risk gene signatures, whereas HMGN2, SP140, CBX5, and SFMBT2 were protective gene signature. Based on the expression levels of seven genes and corresponding coefficients, the risk score was calculated for each osteosarcoma patient as following formula: $Risk score= 0.457\times PSIP1+0.428\times CHD2+0.279\times SMARCA4-0.266\times HMGN2-0.447\times SP140-0.577\times CBX5-0.487\times SFMBT2$. This risk model was validated in the TARGET cohort and GSE21257 cohort. In the TARGET database, the high risk group had 48 patients and low risk group had 47 patients. The Kaplan–Meier curve plot showed that the low-risk group had better clinical outcomes compared with the high-risk group (Fig. 5D, P < 0.001). The predictive accuracy of the risk model in the TARGET cohort was assessed by 1-, 3- and 5-years ROC analysis, of which area under the curve (AUC) values are 0.703, 0.707 and 0.696 (Fig. 5E). Figure 5F–H exhibited the distribution of risk score, survival status and RNA expression profile of selected TFs in the TARGET cohort.

The risk model exhibited similar predictive efficiency in the GSE21257 cohort, where the high risk group had 30 patients and the low risk group had 23 patients. Low risk group members owned better prognosis compared with the high-risk group (Fig. 5I). The AUC values of 1-, 3- and 5-ROC curves were 0.638, 0.781, and 0.772 (Fig. 5J). The distribution of risk score, survival status and RNA expression profile of 8 TFs were presented in Fig. 5K–M. The risk scores of patients from the TARGET cohort and GSE21257 cohort were presented in Table S11.

### The construction and validation of nomogram

**Figure 6:**
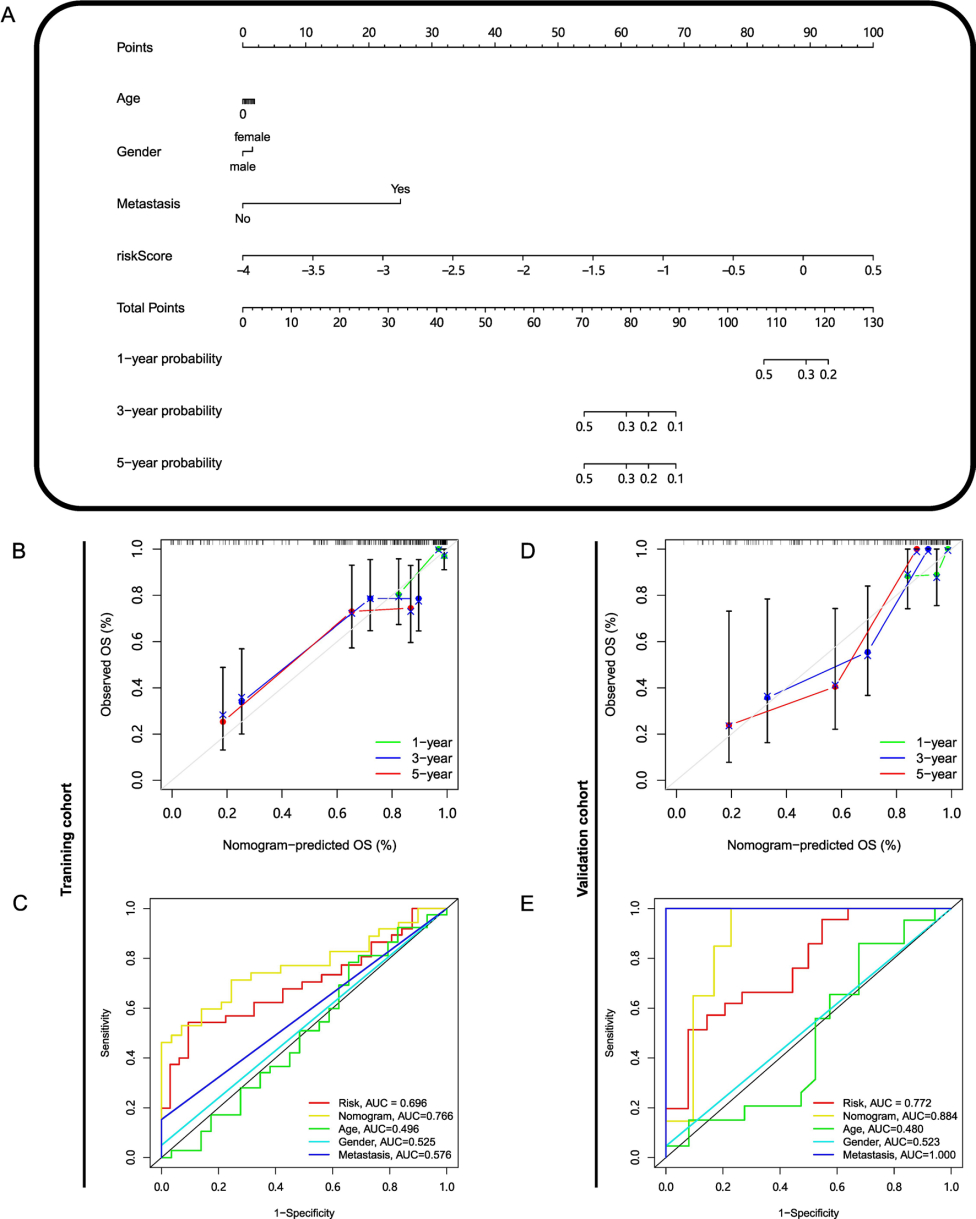
The construction and validation of predictive nomogram. (**A**) The nomogram for predicting 1-, 3-, and 5-year overall survival, was constructed based on age, gender, metastasis, and risk score of patients from the TARGET cohort. Each feature of patients corresponds to a score on the Point scale, and the final score on the Total Points scale results from the addition of the above scores, which corresponds to the survival probability. Point scale: 0–100. The score assigned to each of the characteristics: Age (0.00–1.87); Gender (Male: 0.00; Female: 1.53); Metastasis (No: 0.00; Yes: 25.00); riskScore (0.00–100.00). (**B**) The calibration curve of the nomogram in terms of the agreement between predicted and observed outcomes in the TARGET cohort. (**C**) The time dependent ROC curves of age, gender, metastasis, risk score and nomogram with AUC values of 0.496, 0.525, 0.576, 0.696, and 0.766 at 5 years in the TARGET cohort. (**D**) The calibration of nomogram in the GSE21257 cohort. (**E**) The ROC curves of age, gender, metastasis, risk score and nomogram with AUC values of 0.480, 0.523, 1.000, 0.772, 0.884 at 5 years in the GSE21257 cohort.

The risk score and three clinical features, age, gender, and metastasis status, were incorporated into the construction of the nomogram. Based on the information of TARGET cohort, a nomogram was built, which is capable of predicting the survival probability of 1-, 3-, and 5-years (Fig. 6A). A nomogram is like a rule that can help clinicians quickly estimate the survival probability of a patient. Each characteristic of a patient corresponds to a score on the Points scale, and these scores add up to a final score on the Total Points scale, which corresponds to the survival rate of the patient. TARGET cohort and GSE21257 cohort were used to validate the nomogram. In the TARGET cohort, the calibration curves at 1-, 3-, and 5-years showed outstanding predictive performance (Fig. 6B), and ROC curves at 5-years showed that the AUC values of age, gender, metastasis, risk score, and nomogram were 0.496, 0.525, 0.576, 0.696, and 0.766 respectively (Fig. 6C). In the GSE21257 cohort, the calibration curves at 1-, 3- and 5-years showed good predictive performance as well (Fig. 6D), and the AUC values of ROC curves of age, gender, metastasis, risk score, and nomogram were 0.480, 0.523, 1.000, 0.772, 0.884 (Fig. 6E).

### The role of risk score in clinical characteristics and immune infiltration

**Figure 7:**
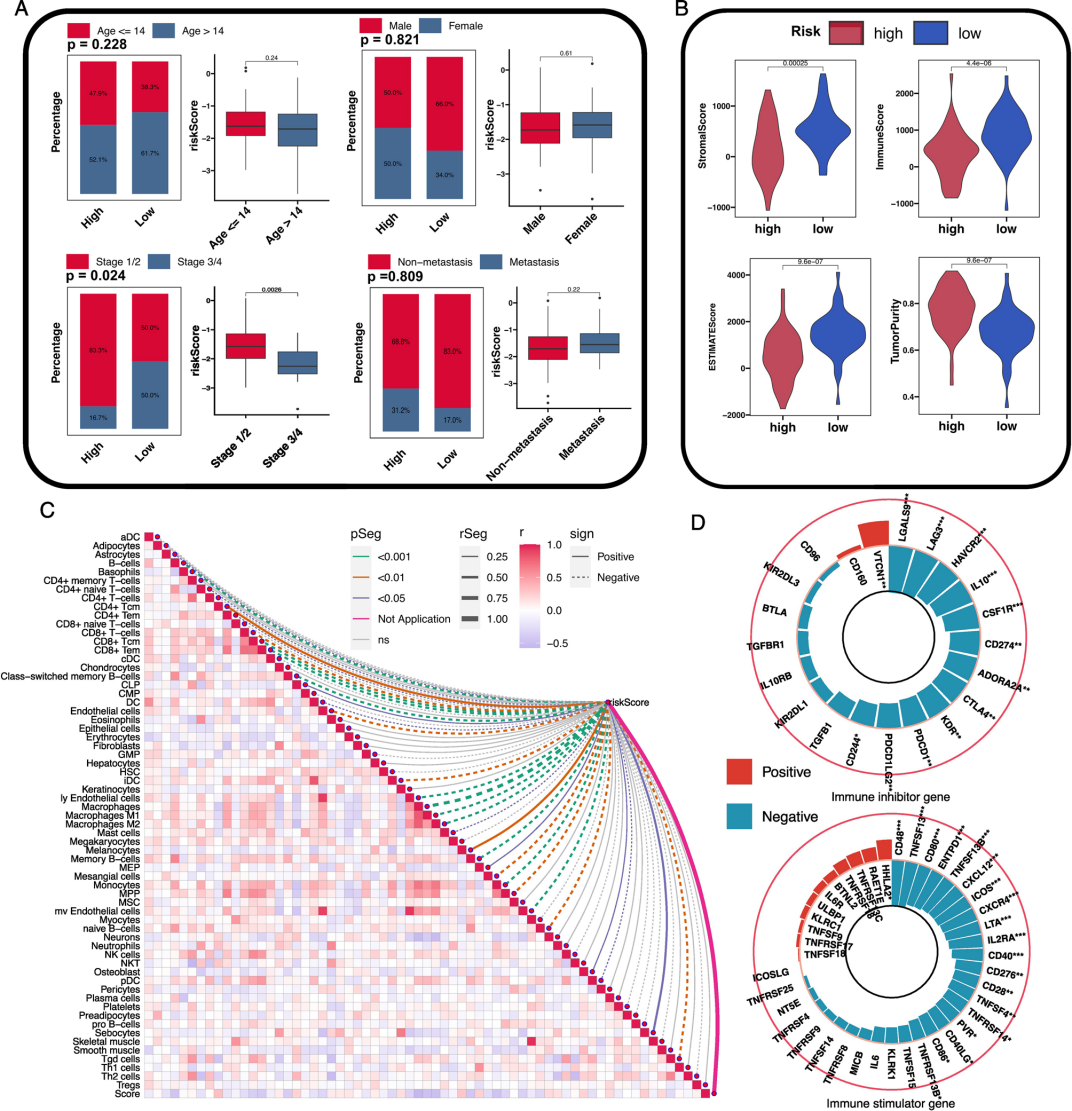
The association of risk score with clinical features and TME. (**A**) The association of risk score with age, gender, histologic response, and metastasis. Histograms presented the distribution of clinical features between the high-risk group and the low-risk group in the TARGET cohort. Boxplots presented the differential analysis for risk scores between different clinical features. (**B**) The differential analysis of stromal score, immune score, ESTIMATE score, and tumor purity between high-risk group and low-risk group. (**C**) The correlations of risk score with 64 stromal cells and immune cells. The r represents the correlation between different cells. The correlation between risk score and immune cells was presented by the characteristics of the line: dotted line represents negative correlation, and the solid line represents positive correlation; The color of the line represents the *P* value; The width of the line represents the correlation value. (**D**) The correlation of risk score with immune inhibitor genes and immune stimulator genes. Red represented a positive correlation, and blue represented a negative correlation. The height of the bar plot represents the correlation value. The inner circle represented a correlation coefficient of − 0.5, and the outer circle represented a correlation coefficient of 0.5. *P < 0.05, **P < 0.01, ***P < 0.001.

Four clinical features, age, gender, histologic response, and metastasis, were incorporated into the following study (Fig. 7A). The distribution of age, gender, and metastasis did not differ between the high-risk group and low-risk group (chi-square test, X-square = 1.453, 0.051 and 0.058 respectively, *P* = 0.228, 0.821 and 0.809 respectively), whereas the percentage of patients with histologic response stage 1/2 was higher in high-risk group (chi-square test, X-square = 5.107, *P* = 0.024). And the risk score of patients with histological response stage 1/2 were higher than those with stage 3/4 (*P* = 0.0026). However, the risk scores of patients did not differ in age, gender, and metastasis status (Wilcoxon test, *P* = 0.24, 0.61, and 0.22 respectively).

Risk score exhibited a strong association with TME. In the TARGET cohort, the stromal score, immune score, and ESTIMATE score were higher in the low-risk group (*P* = 2.5e−4, 4.4e−6 and 9.6e−7 respectively), whereas tumor purity was higher in the high-risk group (*P* = 9.6e−7) (Fig. 7B). The relationship between risk score and 64 stromal cells and immune cells was also investigated respectively in the TARGET cohort. 30 types of cells were identified correlated with risk score (*P* < 0.05), among which Astrocytes, B-cells, CD4+ naïve T-cells, CD4+ Tem, CD8+ T-cells, CD8+ Tcm, CD8+ Tem, Class-switched memory B-cells, DC, Endothelial cells, iDC, ly Endothelial cells, Macrophages, Macrophages M1, Macrophages M2, Mast cells, Memory C-cells, Mesangial cells, Monocytes, MPP, mv Endothelial cells, naïve B-cells, pDC, and Tgd cells were negatively correlated with risk score, whereas CD4+ Tcm, CLP, Melanoccytes, MEP, Osteoblast and Sebocytes were positively correlated with risk score (Fig. 7C). In addition, the expression levels of immune inhibitor genes and immune stimulator genes were associated with risk scores (Fig. 7D). Among immune inhibitor genes, LGALS9 was highly negatively correlated with risk score (r = − 0.478, *P* < 0.001), followed by LAG3 (r = − 0.502, *P* < 0.001) and HAVCR2 (r = − 0.457, *P* < 0.001), and VTCN1 was the only gene positively correlated with risk score (r = 0.267, *P* = 0.009). Among immune stimulator genes, CD48 was the gene most negatively correlated with risk score (r = − 0.506, *P* < 0.001), followed by TNFSF13 (r = − 0.491, *P* < 0.001) and CD80 (r = − 0.459, *P* < 0.001), and HHLA2 was the only gene positively correlated with risk score (r = 0.219, *P* = 0.033). The correlation results of risk score with TME cells and immune related genes were presented in Table S12.

### Prediction of drug sensitivity and response to immunotherapy

**Figure 8:**
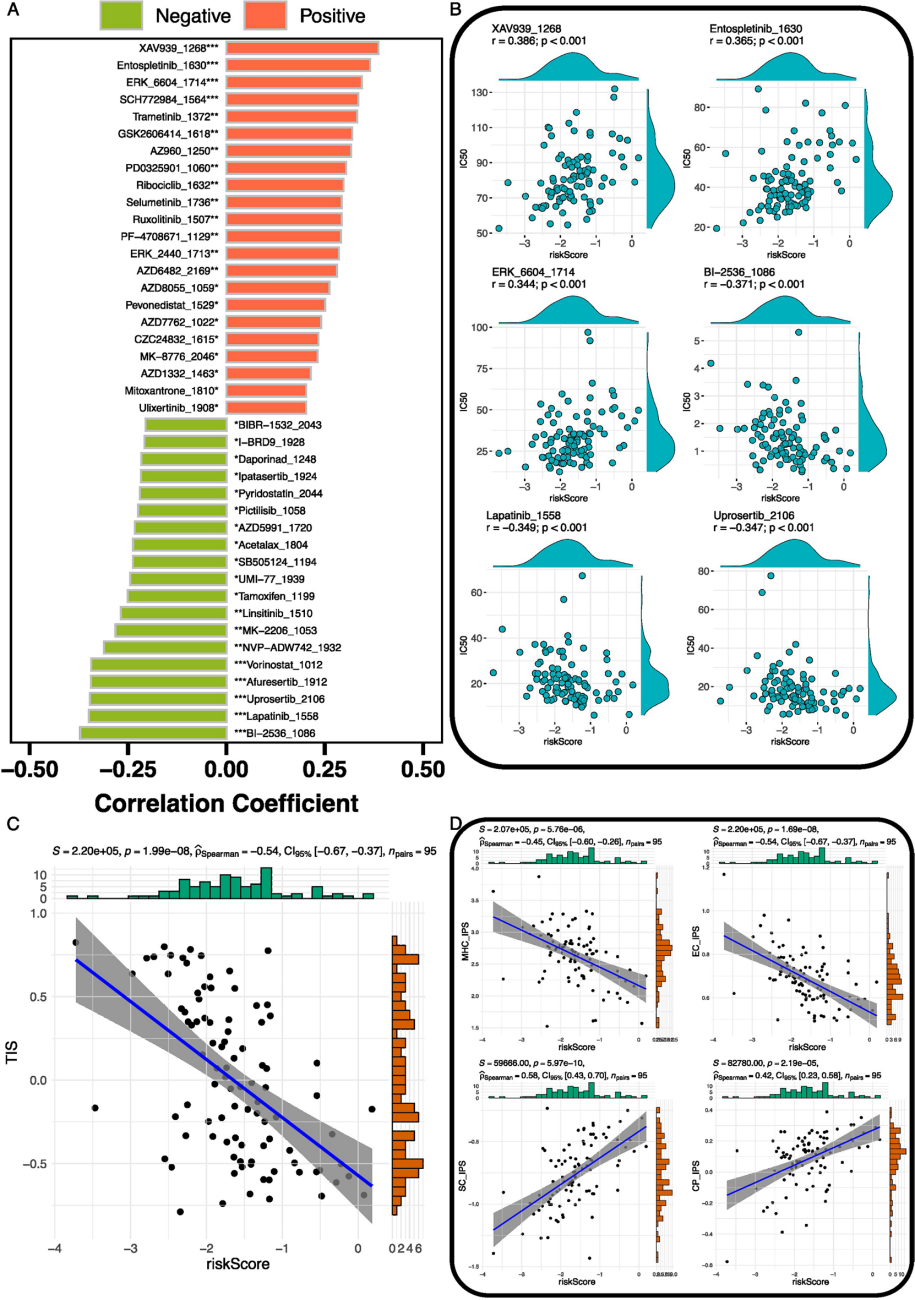
The role of risk score in drug sensitivity and immune therapy. (**A**) The correlation between risk score and drug sensitivity. Red represented a positive correlation, and green represented a negative correlation. (**B**) Scatter charts presented 3 drugs (XAV939, Entospletinib, and ERK_6604) most positively correlated with a risk score and 3 drugs (BI-2536, lapatinib, and Uprosertib) most negatively correlated with risk score. (**C**) The correlation of Tumor Inflammation Signature (TIS) with risk score. (**D**) The correlation of risk score with MHC molecules IPS (MHC_IPS), immunomodulators IPS (CP_IPS), effector cells IPS (EC_IPS), and suppressor cells IPS (SC_IPS). *P < 0.05, **P < 0.01, ***P < 0.001.

Risk score was found to correlate with drug sensitivity and response to immune therapy. The estimated IC50 of drugs is presented in Table S13. In total, 22 drugs were identified positively correlated with risk score, whereas 19 drugs were identified negatively correlated with risk score (Fig. 8A). Figure 8B presented the top 3 drugs (XAV939, Entospletinib, and ERK_6604) positively correlated with the risk score and the top 3 drugs (BI-2536, Lapatinib, and Uprosertib) negatively correlated with the risk score. XAV939 was the drug with the highest positive correlation with risk score (r = 0.386, *P* < 0.001), followed by Entospletinib (r = 0.365, *P* < 0.001), ERK_6604 (r = 0.344, *P* < 0.001). BI-2536 (r = − 0.371, *P* < 0.001) was the drug with the highest negative correlation with risk score, followed by Lapatinib (r = − 0.349, *P* < 0.001) and Uprosertib (r = − 0.347, *P* < 0.001). The result of correlation analysis between drugs and risk score was presented in Table S14. TIS and IPS were used to predict the responses of patients to immunotherapy. TIS were negatively correlated with risk score (r = − 0.54, *P* < 0.001) (Fig. 8C). The correlations between risk and four IPS scores are shown in Fig. 8D. MHC_IPS and EC_IPS were negatively correlated with risk score (r = − 0.45, *P* < 0.001; r = − 0.54, *P* < 0.001), whereas SC_IPS and CP_IPS were positively correlated with risk score (r = 0.58, *P* < 0.001; r = 0.42, *P* < 0.001). The TIS and IPS scores of each osteosarcoma patient are presented in Table S15.

## Discussion

Osteosarcoma is one of the most common malignant childhood tumors occurring in bone tissue. The prognosis of osteosarcoma has not been improved since the 1980s because of the stagnation of treatment methods. Immunotherapy is a novel method using the immune system to attack tumor cells. Given the promising results in clinical trials of many cancers, immunotherapy is expected to lead to a breakthrough in survival. CIC events involve the release of tumor antigens and the infiltration and activation of immune cells, which are highly correlated with the effectiveness of immunotherapy. In addition, a growing number of studies demonstrated the pivotal role of epigenetic modulation in the regulation of immune cells. Therefore, we hypothesize that the cross-talk between CIC and epigenetic modulation is a potential impact factor on the clinical result of immunotherapy, targeting which is a method to overcome the shortcomings of immunotherapy. Both epigenetic modulators and immunotherapy drugs are double-edged swords. The targets of epigenetic drugs occur in tumor tissues as well as normal tissues, and immunotherapy efficacy is also accompanied by drug resistance and irAEs. The combination of epigenetic drugs and immunotherapy is expected to make up for each other’s disadvantages. To advance this combination therapy in osteosarcoma, we explored the molecular signature of epigenetic and immune responses.

The impact of epigenetic changes on CIC events has been identified by numerous studies. In our study, “infiltration of immune cells in tumors” was the CIC event correlated with the most epigenetic factors. In tumor tissues, tumor cells are surrounded by a wide variety of immune cells, such as macrophages, Treg cells, NK cells, B cells etc., which is defined as TME. TME is the soil in which the tumor cells grow, and it is required for the onset and dissemination of osteosarcoma^41^. Through univariate cox analysis, 8 CIC-related epigenetic factors were identified highly correlated with prognosis. SFMBT2, one of the polycomb group proteins, downregulates the expression level of matrix metalloproteinases (MMPs) via interacting with YY1 and various repressive histone marks^42^. What’s more, the downregulation of SFMBT2 was found to advance the infiltration of tumor associated macrophages (TAMs) in prostate cancer^43^. SP140 is an epigenetic reader containing bromodomain, the loss-of-function mutation of which is correlated with multiple autoimmune diseases, such as Crohn’s disease and multiple sclerosis. Recently, SP140 was identified as a repressor of macrophage topoisomerases through a global proteomic strategy^44^. SMARCA4 encodes a transcriptional activator protein called BRG1 that forms the core subunit of the SWI/SNF complex^45^. 20% of human cancers were found accompanied by mutations in subunits of the SWI/SNF complex that has been linked to enhanced interferon response. Besides, the SWI/SNF complex opposes to PRC2 transcriptional repression, whose core enzymatic subunit is EZH2, a histone methyltransferase^46^. Therefore, SMARCA4 is a potential biomarker for evaluating whether to adopt epigenetic modulators or not. SMARCA4 mutation is found in adult-onset epithelial and mesenchymal tumors^47^. A pan-cancer analysis based on the data from TCGA and GTEx database revealed that SMARCA4 was observed upregulated in most cancers, which was correlated with poor overall survival in ACC, MESO, SARC, and SKCM^48^. PSIP1 was identified to control the survival of T cells via its structure changes induced by L-arginine^49^. ACTR6 was found associated with TAMs in lung cancer. Compared with M1 macrophages, ACTR6 was downregulated in M2 macrophages^50^. CBX5 encodes HP1α, a member of the human heterochromatin protein 1 family, which binds H3 di- or tri-methylated at position lysine 9^51^. HP1α also participates in the differentiation and angiogenic function of endothelial progenitor cells through modulating the expression of angiogenic genes and progenitor cell marker genes^52^. CHD2 is required for neural circuit and its mutation is a driver of abnormal brain function, early onset epileptic encephalopathy and intellectual disability^53,54^. In addition, CHD2 mutation was frequently reported in chronic lymphocytic leukaemia and CHD2 was identified essential for myeloid differentiation^55^. HMGN2 protein not only is expressed in tumor cell lines but also work as an anti-tumor effector molecular released by CD8+ T cells^56,57^.

Based on the expression of these eight genes, osteosarcoma was clustered into two subtypes. Function analysis revealed that the two clusters predominantly differed in immune function. The cluster 1 with a better prognosis was enriched with multiple immune-related pathways and GO terms, such as chemokine signaling pathway, Toll-like receptor signaling pathway, Nod like receptor signaling pathway, Natural killer cell mediated cytotoxicity, antigen binding, chemokine activity, activation of immune response, and so on. It was obvious that this clustering was capable of distinguishing the immune characteristics of osteosarcoma patients. Subsequent immune-related analysis also corroborated this result. ESTIMATE score indicated that the osteosarcoma samples in cluster 1 were rich in stromal and immune cells. The higher infiltration level of immune cells is a potential reason for cluster 1 to have a better prognosis. It was found that CD8+ T cell, CD8+ Tem (effector memory T cell), CD8+ Tcm (central memory T cell), B cell, and class-switched memory B cell were enriched in cluster 1. CD8+ T cell is the core of a variety of immunotherapy strategies. Anti-PD1/PDL1 therapy activates CD8+ T cells to attack tumor cells via targeting PD1/PDL1^58^. The high abundance of T cells in cluster 1 suggested that the patients in this cluster were more likely to benefit from immunotherapy. In this study, Macrophage, M1 macrophage, and M2 macrophage were also higher in cluster 1. In addition, M2 macrophages accounted for the vast majority of immune cells in TME of osteosarcoma. M1 macrophages produce pro-inflammatory cytokines, whereas M2 macrophages produce anti-inflammatory cytokines and promote vasculogenesis. The polarization between M1 and M2 macrophages plays a critical role in inflammation and cancer^59^. Besides PD1/PDL1, more and more immune checkpoints emerge and exhibit promising clinical values. Compared with the cluster 2, the cluster 1 had higher expression levels of immune checkpoint genes. The analysis of immune features also demonstrated that the two clusters had great heterogeneity in terms of immunity.

Finally, a prognostic risk model was developed using the LASSO Cox regression algorithm. When the mean squared error was the minimum value, seven genes were identified as significant features, and the coefficient of ACTR6 was zero. Therefore, PSIP1, CHD2, SMARCA4, HMGN2, SP140, CBX5, and SFMBT2 were included in the development of risk model. Although these genes have been linked to various diseases, only a part of them are explored in osteosarcoma. Some studies revealed that HMGN2 plays an anti-tumor role in osteosarcoma. HMGN2 is one of the no-histone nuclear proteins with the most abundance in vertebrates, and its overexpression is able to inhibit cell growth of SaO2 and U2OS cell lines^57^. Another study reported that exogenous HMGN2 protein can inhibit the migration and invasion of osteosarcoma cell lines^60^. Interestingly, CD8+ T cells can release HMGN2 proteins that are transported into tumor cells and induce tumor apoptosis in a dose-dependent manner^56^. It is reported that SMARCA4 is involved in other sarcomas. Small cell carcinomas of the ovary hypercalcemic type (SCCOHT) is characterized by SMARCA4 alterations, and exhibits good response to immunotherapy^45^. The functions of these genes depend on organization and particular cell types, and their roles require investigation through experiments in vivo and in vitro. The patients with the low risk scores in both the train cohort and test cohort owned better prognosis. With the nomogram, clinicians can quickly give patients survival probability. However, the generalization and application of this model in clinical require the inclusion of more training samples in the future to improve accuracy. We found that risk score was mainly correlated with histological response, which indicated that these selected epigenetic factors modulated the response to chemotherapy in osteosarcoma. A recent clinical trial reported that, for patients with localized disease and complete remission after surgery, poor histological response referred to a worse effect of surgery therapy^61^. The investigation into the role of risk score in the TME of osteosarcoma revealed that risk score was a good indicator to predict immune status in the osteosarcoma sample. The risk score was highly negatively associated with CD8+ T cells. Besides, risk score also exhibited a wide correlation with immune modulator genes. Most immune modulator genes were negatively correlated with the risk score, including PDCD1 and CD274. VTCN1 and HHLA2 were the only two immune modulator genes positively correlated with risk score. They are both the members of B7 family. VTCN1, also known as B7-H4, is abnormally upregulated in tumor cells and TAMs, and works as a negative regulatory factor of T cell immune response^62,63^. Song et al.^64^ reported that the inhibition of glycosylation of B7-H4 by NGI-1 improved the immunogenic properties of tumor cells and enhancing the anti-tumor effect of dendritic cells as well as T cells. The role of VTCN1 in the osteosarcoma has been initially investigated by Qiang Dong and Xinlong Ma. They examined the expression level of VTCN1 in osteosarcoma sample by immunohistochemistry, and found that VTCN1 was upregulated in the tumor samples compared with the paired normal tissue samples^65^. HHLA2 is predominantly expressed in various tumor cells and monocytes, but not in normal tissues other than breast, gallbladder, kidney, intestines, and placenta^66^. The role of HHLA2 in cancers is various. In epithelial ovarian cancer, HHLA2 was reported positively correlated with tumor differentiation, the infiltration level of CD8+ T cells, and prognosis^67^. However, in non-small cell lung cancer, HHLA2 deficiency inhibited tumor cell proliferation, migration, and invasion in vitro, and blocked the polarization of M2 macrophages^68^. In osteosarcoma, HHLA2 is upregulated in metastasis tumor samples and associated with worse clinical outcomes^69^. Combining our result and previous studies, we hypothesized that, for osteosarcoma patients without response to anti-PD1/PDL1 therapy, targeting VTCN1 or HHLA2 was a potentially promising treatment method.

Risk score is highly correlated with drug sensitivity. XAV939, a Wnt/β-catenin pathway modulator^70^, was the drug with the highest positive correlation with risk score in this study. It has been confirmed that XAV939 suppresses the proliferation and migration of A549 cells from lung adenocarcinoma in vitro^71^. In osteosarcoma, the blockade of the Wnt/β-catenin pathway by XAV939 reduces the Adriamycin resistance of U2OS cells^72^. It is widely known that chemotherapy resistance is a key issue in the treatment of osteosarcoma. Hence, it is possible that the combination of XAV939 and traditional chemotherapy drugs can enhance the effectiveness of treatment. The drug with the highest negative correlation with risk score was BI-2536, a selective inhibitor of polo-like kinase 1^73^. In vitro and in vivo tests demonstrated that BI-2536 inhibited the proliferation of osteosarcoma cell lines^74,75^. What’s more, BI-2536 was reported to enhance the effects of various conventional chemotherapeutic agents^76–78^. Hence, for patients with the low risk scores, BI-2536 is a potentially potent complement to chemotherapy or neoadjuvant chemotherapy. Beyond drug sensitivity, risk score exhibited a correlation with indicates that are used to predict response to immunotherapy in pan-cancer. Generally, the patients with higher TIS scores benefit more from anti-PD1 therapy^79^. In the current study, the TIS score was highly negatively associated with the risk score, which means that the patients with high risk scores seem to benefit less from immunotherapy. MHC_IPS and EC_IPS were negatively correlated with risk score, whereas SC_IPS and CP_IPS were positively correlated with risk score. It seems like that the TME in patients with high risk scores is deficient in antigen presentation and effector cells. Epigenetic modulation in osteosarcoma is complex and the role of risk score is different in different immunotherapies. Due to the lack of high-quality osteosarcoma immunotherapy cohorts, it is hard to establish a link between scores and response to immunotherapy. It is essential to explore the application of risk scores in the immunotherapy cohorts in the future.

We have to admit that there were several limitations in this study. Firstly, only two datasets were included in this research, and the risk model required validation in more independent datasets. Secondly, epigenetic gene signatures were identified via bioinformatic methods and required experimental validations. Thirdly, because of the lack of an immunotherapy cohort of osteosarcoma, several well-established algorithms were applied to predict immunotherapy response and the role of risk score needs to be explored in real-world data.

## Conclusion

In conclusion, we explored the association between epigenetic factors and CIC events in osteosarcoma. Based on eight factors highly correlated with CIC events, two epigenetic subtypes in osteosarcoma were identified via NMF clustering. The two subtypes were mainly distinguished by immune response and cell cycle regulation. Finally, a clinical risk model and a nomogram were established, which can help clinicians quickly predict the survival probability of patients. Risk score is strongly correlated with drug sensitivity, immune infiltration, and immune checkpoint genes. Our study could shed a novel light on the epigenetic modulation mechanism of osteosarcoma and helps search for potential novel drugs.

### Supplementary Information

Supplementary Legends.

Supplementary Table S1.

Supplementary Table S2.

Supplementary Table S3.

Supplementary Table S4.

Supplementary Table S5.

Supplementary Table S6.

Supplementary Table S7.

Supplementary Table S8.

Supplementary Table S9.

Supplementary Table S10.

Supplementary Table S11.

Supplementary Table S12.

Supplementary Table S13.

Supplementary Table S14.

Supplementary Table S15.

## Data Availability

The gene expression data and corresponding clinical information were collected from public database, including TARGET cohort (https://www.cancer.gov/ccg/research/genome-sequencing/target) and GSE21257 cohort (https://www.ncbi.nlm.nih.gov/geo/query/acc.cgi?acc=gse21257). The EpiFactor were collected from EpiFactors Database (https://epifactors.autosome.org/).

## Abbreviations

FDA: Food and Drug Administration
GEO: Gene Expression Omnibus
GDSC: Genomics of Drug Sensitivity in Cancer
KEGG: Kyoto encyclopedia of genes and genomes
GO: Gene ontology
CC: Cellular component
MF: Molecular function
BP: Biological process
NMF: Non-negative matrix factorization
LASSO: Least absolute shrinkage and selection operator regression
GSVA: Gene set variation analysis
GSEA: Gene set enrichment analysis
ssGSEA: Single sample gene set enrichment analysis
IC50: Half maximal inhibitory concentration
PCA: Principal component analysis
AUC: Area under the curve
ROC: Receiver operating characteristic curve
OS: Osteosarcoma
TIS: Tumor inflammation signature score
IPS: Immunophenoscore
MHC_IPS: MHC molecules IPS
CP_IPS: Immunomodulators IPS
EC_IPS: Effector cells IPS
SC_IPS: Suppressor cells IPS
PDL: Programmed death ligand
ICB: Immune checkpoint blockade
irAEs: Immune-related adverse events
CIC: Cancer immunity cycle
TME: Tumor microenviroment
lncRNA: Long noncoding RNA
ICD: Immunogenic cell death
MMPs: Matrix metalloproteinases
TAMs: Tumor associated macrophages
SCCOHT: Small cell carcinomas of the ovary hypercalcemic type
DC: Dendritic cells
MPP: Multipotent rogenitors
CD8+ Tem: CD8+ effector memory T-cells
CMP: Common myeloid progenitors
GMP: Granulocyte–macrophage progenitors
MEP: Megakaryocyte–erythroid progenitors
Tregs: Regulatory T-cells
CD4+ Tcm: CD4+ central memory T-cells
mv Endothelial cells: Microvascular endothelial cells
CD4+ Tem: CD4+ effector memory T-cells
CD8+ Tcm: CD8+ central memory T-cells
ly Endothelial cells: Lymphatic endothelial cells
MSC: Mesenchymal stem cells
aDC: Activated dendritic cells
cDC: Xonventional dendritic cells
pDC: Plasmacytoid dendritic cells
