# Supplementary material for: A CIC-related-epigenetic factors-based model associated with prediction, the tumor microenvironment and drug sensitivity in osteosarcoma
Source: Sci Rep. 2024 Jan 15;14:1308. doi: 10.1038/s41598-023-49770-2 (PMC10789798; doi:10.1038/s41598-023-49770-2)
Supplement: Supplementary file 1 — Supplementary Legends. [file 41598_2023_49770_MOESM1_ESM.docx]

| Supplementary file | Title |
| --- | --- |
| Table S1 | Epigenetic factor gene list. |
| Table S2 | The RNA expression data of TARGET OS cohort and GSE21257 cohort. |
| Table S3 | The clinical information of TARGET OS cohort and GSE21257 cohort. |
| Table S4 | Cancer immunity cycle score. |
| Table S5 | The correlation between cancer immunity cycle and epigenetic factors. |
| Table S6 | The CIC-related epigenetic factors with prognostic value. |
| Table S7 | Molecular subtypes using NMF algorithm. |
| Table S8 | GSVA result. |
| Table S9 | GSEA result. |
| Table S10 | The result of immune infiltration analysis. |
| Table S11 | The risk score of clinical risk model. |
| Table S12 | The correlation between risk score and TME cells and immune related genes. |
| Table S13 | Predicted IC50 using oncoPredict R package. |
| Table S14 | The correlation between drug sensitivity and risk score. |
| Table S15 | TIS score and IPS score. |
